# A High-Precision Identification Method for Maize Leaf Diseases and Pests Based on LFMNet under Complex Backgrounds

**DOI:** 10.3390/plants13131827

**Published:** 2024-07-03

**Authors:** Jintao Liu, Chaoying He, Yichu Jiang, Mingfang Wang, Ziqing Ye, Mingfang He

**Affiliations:** 1 College of Electronic Information & Physics, Central South University of Forestry and Technology, Changsha 410004, China; 20212553@csuft.edu.cn (J.L.); t20040528@csuft.edu.cn (C.H.); t20060566@csuft.edu.cn (M.W.); 2 Hunan Polytechnic College of Environment and Biology, Hengyang 421005, China; jiangyichu@hnebp.edu.cn

**Keywords:** identification of maize leaf diseases and pests, LFMNet, LMSB, FLB, MLFFA

## Abstract

Maize, as one of the most important crops in the world, faces severe challenges from various diseases and pests. The timely and accurate identification of maize leaf diseases and pests is of great significance for ensuring agricultural production. Currently, the identification of maize leaf diseases and pests faces two key challenges: (1) In the actual process of identifying leaf diseases and pests, complex backgrounds can interfere with the identification effect. (2) The subtle features of diseases and pests are difficult to accurately extract. To address these challenges, this study proposes a maize leaf disease and pest identification model called LFMNet. Firstly, the localized multi-scale inverted residual convolutional block (LMSB) is proposed to perform preliminary down-sampling on the image, preserving important feature information for the subsequent extraction of fine disease and pest features in the model structure. Then, the feature localization bottleneck (FLB) is proposed to improve the model’s ability to focus on and locate disease and pest characteristics and to reduce interference from complex backgrounds. Subsequently, the multi-hop local-feature fusion architecture (MLFFA) is proposed, which effectively addresses the problem of extracting subtle features by enhancing the extraction and fusion of global and local disease and pest features in images. After training and testing on a dataset containing 19,451 images of maize leaf diseases and pests, the LFMNet model demonstrated excellent performance, with an average identification accuracy of 95.68%, a precision of 95.91%, a recall of 95.78%, and an F1 score of 95.83%. Compared to existing models, it exhibits significant advantages, offering robust technical support for the precise identification of maize diseases and pests.

## 1. Introduction

Maize is an important crop widely cultivated, not only for its high nutritional value but also as an important food source for humans and animals [1]. It also plays an important role in the field of food processing and can be used to produce edible oil, animal feed, maize flour, beer, and other products [2]. Maize is also a key raw material in many industrial production processes [3]. Since 2001, maize has surpassed rice to become the world’s second-largest grain. It is estimated that maize, as the staple food for over 200 million people, provides about 15% of the world’s protein and 20% of calories [4]. Therefore, the impact of maize diseases and pests on maize yield and the resulting economic losses cannot be ignored [5]. The accurate identification of diseases and pests on maize leaves and the corresponding prevention and control measures are crucial for reducing economic losses caused by diseases and pests during the growth process. At present, the identification of maize diseases and pests mainly relies on manual work, and preliminary diagnosis is made through people’s learning and understanding of the characteristics of maize leaf diseases and pests. However, this method has problems of low efficiency, susceptibility to subjective factors such as emotions, fatigue, etc., and requires a large amount of labor, resulting in high costs and difficulty in large-scale application. In order to effectively reduce the impact of diseases and pests on maize growth and yield, it is necessary to diagnose the diseases and pests as early as possible and take corresponding prevention and control measures, such as spraying pesticides, increasing fertilizer application, and introducing natural enemies of pests. This requires the precise identification of maize leaves to ensure the adoption of correct treatment methods and an effective response to the challenges posed by maize diseases and pests.

With the rapid development of computer vision technology, especially driven by deep learning applications such as image segmentation, classification, and detection, significant progress has been made in the field of automated identification of plant diseases and pests [6]. By using deep learning models to accurately identify the disease and pest characteristics of plant leaves, not only can the accuracy of diagnosis be improved, but early warning and timely intervention can also be achieved, effectively reducing the impact of diseases and pests on plant growth and yield. Nivethithaa and Vijayalakshmi (2023) proposed an optimized fish swarm optimization-support vector machine (FSOSVM) model for plant disease identification [7]. This model achieved an accuracy of 98.3% and 98.9% in identifying rice and maize leaf diseases, respectively. However, the FSOSVM model only identifies four types of maize leaf diseases, which may not meet the needs of diversified disease detection in actual agricultural production. Ramadan et al. (2023) used various convolutional neural networks and transformer architectures to detect maize leaf diseases, among which the ViT-B/16 model performed the best, with an accuracy of 94.51% [8]. However, compared to CNN, the ViT model has higher computational resource requirements, which may lead to insufficient identification efficiency in practical applications. Mehta et al. (2023) constructed and evaluated a federated learning CNN model for identifying and diagnosing maize diseases, achieving an overall accuracy of 89.4% on the test set and demonstrating adaptability and consistency to various disease categories [9]. However, 89.4% of the identification accuracy may not meet the requirements for precise identification of maize leaf diseases and pests in practical applications.

Bhuyan et al. (2023) proposed a Res4net-CBAM model that combines the Res4net architecture and the Convolutional Block Attention Module (CBAM) [10]. This model can extract complex features related to different diseases and achieve an average identification accuracy of 98.27% on a self-built tea disease dataset. Although Res4net CBAM has advantages in disease feature extraction, further optimization may be needed to deal with complex background interference in practical applications. Lv et al. (2020) integrated maize leaf disease feature enhancement technology with the DMS-Robust Alexnet approach to propose a novel method for maize disease identification [11]. This method achieved a classification accuracy of 98.62% on a dataset containing images of six types of maize leaves. However, the feature extraction ability of DMS-Robust Alexnet may still need to be improved when facing a wider variety of maize diseases and pests. Lin et al. (2021) designed a novel neural network EM-ERNet based on the ResNet backbone architecture and a particle swarm optimization ELM algorithm (PELM) to accelerate the fusion network of the final fully connected layer, achieving excellent identification performance on public banana disease detection datasets [12]. However, considering that banana images in public datasets have already undergone background processing in advance, this may limit the model’s adaptability in real-world environments.

**Figure 1:**
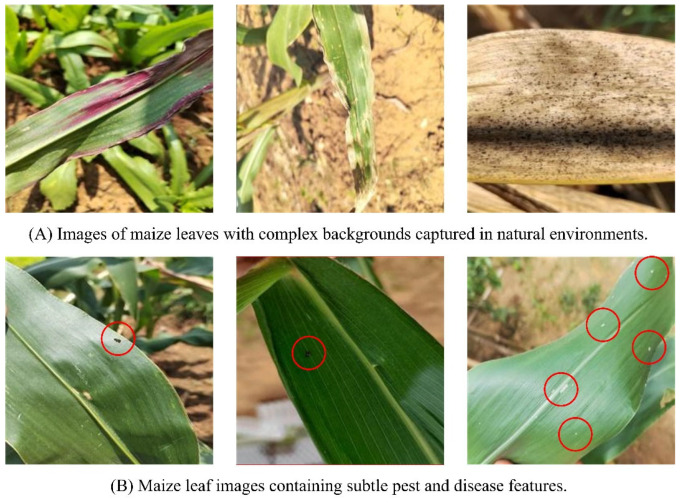
Difficulties in maize leaf disease and pest identification. The red circles in the figure indicate the subtle features of maize leaves.

These studies provide valuable experience for identifying maize leaf diseases and pests, but the identification process still faces some challenges:

(1) Complex background interference: Images captured in natural environments may have various complex backgrounds, as shown in Figure 1A. These complex backgrounds may affect the model’s learning of disease and pest features, leading to the model capturing features unrelated to diseases and pests, thereby reducing identification accuracy.
(2) The characteristics of diseases and pests are subtle: Some diseases and pests have relatively small characteristics on maize leaves, as shown in Figure 1B. These subtle features occupy a small proportion of pixels in the entire image, making feature extraction challenging.

In order to solve the problem of complex background interference, Qian et al. (2022) proposed a model for maize leaf identification based on converters and self-attention [13]. The model represents the visual information of local regions in the image through labels, calculates the correlation between local regions using attention mechanisms, and finally integrates global information for classification, effectively reducing the interference of complex backgrounds. Chen et al. (2020) used binary wavelet transform combined with Retinex (BWTR) for image denoising and enhancement and used the artificial bee colony algorithm (ABCK)-optimized KSW to separate tomato leaves from the background [14]. Finally, a dual-channel residual attention network model (B-ARNet) was used for image identification, with an overall identification accuracy of approximately 89%. Fang et al. (2022) proposed the HCA-MFFNet model, which uses the Hard Coordination Attention Mechanism (HCA) at different spatial scales to extract features of maize leaf disease images, reducing the impact of complex backgrounds [5]. The model achieved an average identification accuracy of 97.75% and an F1 score of 97.03% on a self-built dataset. Zhang et al. (2023) proposed BCTNet for detecting apple leaf diseases in unconstrained environments. In BCTNet, a Cross-Attention Module (CAM) is adopted to reduce the computational workload of the detection network in non-disease areas in order to reduce the impact of background interference information on the network’s feature representation ability [15]. BCTNet achieved an accuracy of 85.23% and an average detection speed of 33 FPS on a self-built dataset.

In order to address the issue of subtle features, Alirezazadeh et al. (2022) applied the Convolutional Block Attention Module (CBAM) to the output feature map of CNN, highlighting important local regions and extracting more discriminative features [16]. This enabled EfficientNetB0 + CBAM to achieve a classification accuracy of 86.89% on the pear-leaf-disease dataset, DiaMOS Plant. Zhang et al. (2021) proposed an apple disease detection method based on a deep multi-scale dual-channel convolutional neural network (DMCNN) [17]. The HSV color subspace analysis network and RGB color subspace texture analysis network are used to extract features, and a DMCNN was constructed using a homologous feature cross-fusion mechanism. A detection rate of 99.5% was achieved on a self-built apple disease dataset. Li et al. (2023) proposed a tomato-leaf-disease identification method based on LMBRNet, constructed a Comprehensive Grouping Discriminant Residual (CGDR), and utilized the multi-branched structure of CGDR to capture diverse feature information about tomato leaf diseases in different dimensions and receptive fields, achieving an accuracy rate of 99.7% in tomato leaf disease identification [18]. Deng et al. (2023) proposed a novel image identification network, GR-ARNet, based on ResNet50, which implements the Ghost Module to improve the network’s ability to extract deep feature information about banana leaf diseases and identification speed [19]. The ResNeSt module is also used to adjust the weights of each channel, enhance the banana disease feature extraction ability, and effectively reduce the identification error rate of similar diseases. The GR-ARNet model achieved an average accuracy of 96.98% and an accuracy of 89.31%.

These studies indicate that by combining advanced image processing techniques, attention mechanisms, multi-scale analysis, feature fusion, and innovative deep learning architectures, the accuracy and robustness of maize leaf disease and pest identification can be effectively improved. In order to further address the issues of complex background interference and subtle disease and pest characteristics in the identification of maize leaf diseases and pests, this study proposes an LFMNet model based on ResNext50. The following are the main contributions of this article:

(1) Innovative design of the LFMNet model:
  (a) A localized multiscale inverted residual convolutional block (LMSB) was proposed, which overcomes the problem of losing important features during initial down-sampling in traditional convolution by combining local attention mechanisms, inverted residual structures, and multi-scale feature extraction. It achieves image down-sampling while retaining important disease and pest feature information for accurate extraction of fine disease and pest features in the subsequent structure of the model.
  (b) The feature localization bottleneck (FLB) was proposed, which effectively combines the bottleneck module with an attention mechanism to address the shortcomings of the bottleneck module in dealing with complex background interference and extracting subtle features. The FLB helps the model focus on the disease and pest feature areas in the feature map, reducing the interference of complex backgrounds and helping to accurately extract subtle disease and pest features, thereby improving the accuracy and robustness of the model for disease and pest identification.
  (c) The multi-hop local-feature fusion architecture (MLFFA) was proposed, which effectively overcomes the shortcomings of ResNext50 in fine feature extraction through a multi-hop local-feature fusion architecture. The combination of Efficient Localized Convolution (ELC) and a multi-stage feature fusion strategy significantly improves the model’s ability to capture and integrate subtle features of maize leaf diseases and pests, achieving more accurate disease and pest identification performance.
(2) On the self-built dataset, the LFMNet model achieved an average testing accuracy of 95.68% and an F1 score of 95.83%, demonstrating excellent performance. The LFMNet model has significant advantages over existing methods, effectively reducing the interference of complex backgrounds in practice and achieving the accurate extraction of fine features of diseases and pests. LFMNet can not only accurately identify maize leaf diseases and pests but may also provide reference values for the identification of other crop diseases and pests.

## 2. Maize Leaf Diseases and Pests Characteristics

The growth and yield of maize are not only affected by weed competition but may also be threatened by various pests and pathogens, as well as the effects of deficiencies in trace elements such as phosphorus and potassium.

Maize diseases include northern leaf blight, gray leaf spot, common rust, blight, and mold, as well as phosphorus and potassium deficiency. Northern leaf blight is caused by helminthosporium turcicum, which leads to slender gray-green to brown spots on leaves [20]. Gray leaf spot is caused by cercospora zeae-maydis and cercospora zeina [21]. The disease is initially surrounded by a yellow halo and later forms rectangular gray spots parallel to the leaf veins. Common rust causes orange-brown abscesses to appear on the leaves, which, in severe cases, cover the entire leaf surface [22]. Blight is caused by the individual or combined infection of several types of fusarium or pythium, with grayish green leaves that resemble hot water or frost. Mold is caused by infection by mold or miscellaneous fungi, with black mold spots on the leaves. When phosphorus deficiency occurs, the leaf tips and edges appear purple-red, while when potassium deficiency occurs, yellow stripes appear on the leaves, from the leaf tips to the base.

Maize pests include the leaf beetle, the red spider mite, the *Mythimna separata* (walker), and the *Phyllotreta striolata*. After being damaged by the leaf beetle, there are residual network veins or epidermis on the leaves, which appear as small, irregular white spots from a distance [23]. The red spider mite causes the surface of leaves to be covered with flocculent or mesh-like substances [24]. The damage caused by the *Mythimna separata* (walker) leads to holes or irregular notches in the leaves [25]. The *Phyllotreta striolata* causes adult insects with yellow stripe markings to be visible on the leaves [26].

Table 1 provides a detailed description of the leaf characteristics of these 11 maize diseases and pests, providing important references for the identification and management of diseases and pests.

## 3. Materials and Methods

### 3.1. Data Acquisition

This study constructed a comprehensive dataset of maize leaf diseases and pests, named MLDPs. This dataset consists of two parts: one part is sourced from images of maize leaf diseases in the Plant Village database [27], including symptoms of gray leaf spot, common rust, and northern leaf blight; the other part is the images of maize diseases and pests obtained through on-site photography, covering symptoms of eight diseases and pests such as blight, potassium deficiency, phosphorus deficiency, and mold, as well as leaf beetle, red spider mite, *Mythimna separata* (walker), and *Phyllotreta striolata*, as shown in Table 2. During the data collection process, we used a mobile phone camera to capture and store images of maize leaves affected by diseases and pests. The device was a Huawei Honor 50. When taking photos, the distance between the mobile phone camera and the leaf was 10–50 cm, and the focal length was about 1.8. With the assistance of agricultural experts, we rigorously screened and classified the photos of maize diseases and pests we captured, excluding images of fewer types of diseases and pests. After screening, we obtained eight clearly classified images of maize diseases and pests. In order to integrate these two parts of the data, we combined three types of maize disease leaf images and healthy leaf images from Plant Village with eight types of maize disease and pest images captured on site. Specifically, the images of the Plant Village account for 27.74% of the dataset, while the images we captured ourselves account for 72.26%. Considering the pixel differences between the on-site captured images and the images in the Plant Village database, we resized all images to 256 × 256 pixels to ensure consistency in the dataset. The final dataset was not only more diverse in image types, covering various symptoms of maize diseases and pests, but also underwent strict screening and standardization in image quality, providing high-quality data support for the subsequent automatic identification and analysis of maize leaf diseases and pests. The specific number of various images in the initial dataset is shown in Table 3, which provides an important reference for evaluating and comparing the performance of different disease and pest identification models.

**Figure 2:**
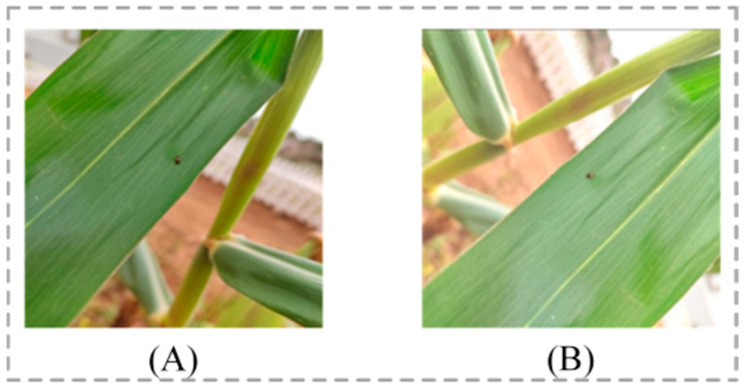
Comparison of images before and after data augmentation: (**A**) an image before data augmentation; (**B**) the image after data augmentation.

We noticed that the number of gray leaf spot, potassium deficiency, and *Phyllotreta striolata* images in the MLDP dataset is relatively small, which may affect the balance of model training and identification accuracy. To address this issue, we implemented data augmentation techniques on these three types of images to increase their sample size. The data augmentation methods we use include image rotation and brightness changes, which not only effectively expand the number of images but also improve the model’s generalization ability for images under different environmental conditions, as shown in Figure 2. Through these enhancement methods, we can simulate the appearance of maize leaf diseases and pests under different angles and lighting conditions, thereby providing more diverse training samples for the model. The specific distribution of the number of enhanced images is shown in Table 4.

### 3.2. LFMNet

**Figure 3:**
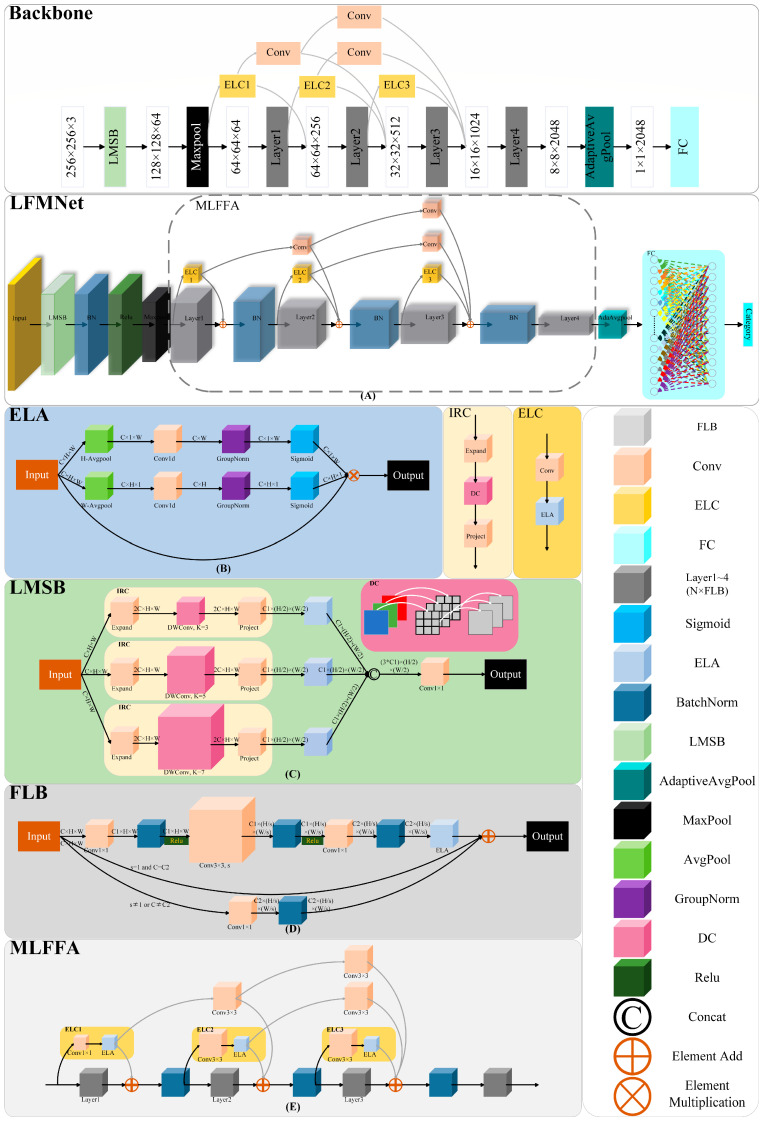
The network structure diagrams of the LFMNet and its constituent modules: (**A**) LFMNet; (**B**) ELA; (**C**) LMSB; (**D**) FLB; (**E**) MLFFA.

In this study, we propose an LFMNet based on ResNext50 [28], aimed at accurately identifying maize leaf diseases and pests in the natural environment. LFMNet combines the LMSB, the FLB, and the MLFFA. Through these improvements, LFMNet has improved the accuracy of identifying maize leaf diseases and pests, ensuring superior identification performance in natural environments, overcoming the interference of complex backgrounds during identification, and effectively extracting subtle disease and pest features. The overall structure of the LFMNet is shown in Figure 3A.

The LFMNet includes the LMSB, four layers composed of the FLB, and the MLFFA. Firstly, the LMSB is used for initial down-sampling and feature extraction of the image, followed by dimensionality reduction and feature enhancement using the max pooling layer. Then, using the MLFFA to capture and fuse subtle features of the image, its multi-hop local-feature fusion strategy effectively overcomes the limitations of the original model in extracting subtle disease and pest features. The workflow of the MLFFA is as follows:

The input image is first sent to both Layer1 and ELC1 for feature extraction, and the output results of Layer1 and the ELC1 module are added element by element. After batch normalization, they are used as inputs to Layer2 and ELC2. Afterwards, the output of ELC1 after one convolution is added, element by element, to the output of Layer2 and the ELC2 module, and batch-normalized as the input to Layer3. Then, the output of ELC1 after two convolutions is added, element by element, to the output of Layer3, the output of ELC2 after one convolution, and the output of ELC3. After batch normalization, it is used as the input to Layer4. The output from Layer4, which is also the output of the MLFFA, undergoes dimensionality reduction via an average pooling layer. This reduced representation is then transformed into a one-dimensional vector by the flattening operation and subsequently passed to a fully connected layer for the final output of image identification results.

#### 3.2.1. Efficient Local Attention (ELA)

In deep learning, attention mechanisms allow models to selectively focus on certain parts of input data while ignoring other irrelevant information. This can improve the model’s perception ability of key information and reduce the interference of noise or irrelevant information. Therefore, we introduced the ELA attention mechanism to enhance the subtle features of diseases and pests in the image, improve the model’s localization ability for these subtle features, and reduce attention to complex backgrounds in the image [29]. The structure of the ELA attention mechanism is shown in Figure 3B.

In terms of spatial dimension, the ELA module adopts strip pooling [30] to obtain feature vectors in the horizontal and vertical directions, respectively. This method uses narrower convolution kernels to capture remote dependencies, avoiding the impact of irrelevant regions on label prediction and thus obtaining rich target position features in their respective directions.

For the input image (with shapes C, H, and W), first perform average pooling along the horizontal and vertical directions of the image to generate the outputs of C channels with a height of H (C × H × 1) and C channels with a width of W (C × 1 × W), represented as $z_{h}$ and $z_{w}$, respectively, as shown in Equations (1) and (2).

(1) $$z_{h}=\frac{1}{H}\sum_{0\leq i<H}x_{c}(h,i)$$

(2) $$z_{w}=\frac{1}{W}\sum_{0\leq i<W}x_{c}(j,w)$$

where $z_{h}$ and $z_{w}$ capture the global sensory field and precise positional information. In order to enhance the horizontal and vertical position information, one-dimensional convolutions are applied to $z_{h}$ and $z_{w}$, respectively. This type of convolution is more suitable for processing sequential signals and has higher computational efficiency. The size of the 1D convolution kernel can be adjusted to indicate the coverage range of local interactions. Subsequently, the enhanced position information was processed using the group normalization (GN) [31] and sigmoid activation functions to obtain the horizontal and vertical position attention representations $y^{h}$ and $y^{w}$, as shown in Equations (3) and (4).

(3) $$y^{h}=\sigma (GN\left({Conv}_{1\times 1}\left(z_{h}\right)\right))$$

(4) $$y^{w}=\sigma (GN\left({Conv}_{1\times 1}\left(z_{w}\right)\right))$$

In the above description, the nonlinear activation function is represented as σ and 1D convolution is represented as Conv (1 × 1). The convolution kernel of Conv (1 × 1) can be set to 5 or 7, and the group can be set to C or (C/8).

Finally, by performing element-wise multiplication between the input image $x_{c}$ and the positional attention representations $y^{h}$ and $y^{w}$, the output Y of the ELA module is obtained, as shown in Equation (5).

(5) $$Y=x_{c}\times y^{h}\times y^{w}$$

As an important component of LFMNet, the ELA module provides efficient feature enhancement and localization capabilities for the model, which helps to improve the accuracy and robustness of maize disease and pest identification.

#### 3.2.2. Localized Multi-Scale Inverted Residual Convolutional Block (LMSB)

In deep learning, the initial design of the network structure has a significant impact on subsequent feature extraction and model performance. The ResNext50 uses a large convolution kernel of 7 × 7 in the initial stage for the initial down-sampling and feature extraction of images. This design can provide a larger receptive field, but it may also overlook some subtle features in the image, especially when dealing with images such as maize diseases and pests that are easily affected by complex backgrounds.

Therefore, we propose a localized multi-scale inverted residual convolutional block (LMSB) to replace the 7 × 7 convolution kernel for more precise initial down-sampling and feature extraction of images. The structure of the LMSB is shown in Figure 3C.

The LMSB consists of two parts: IR2, based on the inverted residual block [32], and ELA. IR2 contains three convolution operations: expand convolution, depth-wise convolution, and project convolution, with related parameters c1, c2, stripe, k, p, and e, where e is the expansion factor. After receiving the input image, IR2 first expands the number of channels through expansion convolution, as shown in Equation (6). Among them, $y_{expand}$ is the output after expanding convolution, x is the input feature map, and ${conv}_{1\times 1}$ represents a 1 × 1 convolution operation.

(6) $$y_{expand}=\left\{\begin{array}{cc} x, & \;e\leq 1\\ {conv}_{1\times 1}(x), & \;e>1 \end{array}\right.$$

Next, use depth-wise convolution to convolve and obtain the feature map. Finally, the project convolution down-samples to obtain a half-sized output image, as shown in Equation (7). DC represents depth-wise convolution, and PC represents project convolution.

(7) $${out}_{IR2}=PC(DC\left(y_{expand}\right))$$

The LMSB contains IR2 with three different convolution kernel sizes (3, 5, and 7), which perform feature extraction on the input image in parallel, as shown in Equation (8). x is the input image, where ${IR2}_{3\times 3}$, ${IR2}_{5\times 5}$, and ${IR2}_{7\times 7}$ represent IR2 with convolution kernels of 3, 5, and 7, respectively.

(8) $$\left\{\begin{array}{c} \mathrm{out}1={IR2}_{3\times 3}\left(x\right)\\ \mathrm{out}2={IR2}_{5\times 5}\left(x\right)\\ \mathrm{out}3={IR2}_{7\times 7}\left(x\right) \end{array}\right.$$

Each output image of IR2 is then subjected to the ELA attention mechanism to obtain richer feature information, as shown in Equation (9).

(9) $$\left\{\begin{array}{c} \mathrm{out}1^{\prime }=\mathrm{ELA}\left(out1\right)\\ \mathrm{out}2^{\prime }=\mathrm{ELA}\left(out2\right)\\ \mathrm{out}3^{\prime }=\mathrm{ELA}\left(out3\right) \end{array}\right.$$

Finally, three feature maps processed by ELA are concatenated, and dimensionality reduction and feature fusion are performed using a 1 × 1 ordinary convolution to obtain the final output image, as shown in Equation (10).

(10) $$out={{conv}_{1\times 1}}^{\prime }(concat\left(out1^{\prime },out2^{\prime },out3^{\prime }\right),dim=1)$$

The multi-scale and localized design of the LMSB enables it to simultaneously capture micro details and macro structures in images, focusing on disease and pest feature areas, thus outperforming convolution with a 7 × 7 large kernel in initial down-sampling and feature extraction. This design helps to improve the identification rate of the model for maize disease and pest images, especially in situations where complex backgrounds and subtle features coexist in natural environments.

#### 3.2.3. Feature Localization Bottleneck (FLB)

Bottleneck is an important component module in the ResNext50 model. This module improves the training efficiency of deep networks through efficient parameter utilization and residual connections. However, when dealing with the task of identifying maize diseases and pests, the bottleneck module may face some challenges, especially under the interference of complex backgrounds. The bottleneck module may not be sufficient to handle complex textures and patterns present in the background, which may interfere with the model’s judgment and affect identification accuracy. To address this issue, this study proposes the feature localization bottleneck (FLB) module to enhance the identification performance of the LFMNet model. The network structure of FLB is shown in Figure 3D.

FLB preserves the residual connection characteristics of bottleneck and enhances feature representation by combining localized attention mechanisms and group convolution. This module first utilizes point-to-point convolution to reduce the number of channels and reduce the computational complexity of subsequent group convolutions. The output of this step is represented as ${out}_{1}$, as shown in Equation (11).

(11) $${out}_{1}={PointwiseConv}_{1}(x)$$

After dimensionality reduction, the feature map is input into group convolution through batch normalization (BN) [33] and the relu activation function. Group convolution extracts spatial features without changing the number of channels, and each group learns different features to enhance the model’s representation ability. The output of this step is represented as ${out}_{2}$, as shown in Equation (12).

(12) $${out}_{2}=GroupConv(Relu(BN({out}_{1}))$$

The feature maps that have undergone group convolution are further subjected to batch normalization and the relu activation function, and then input into another point-by-point convolution for dimensionality enhancement. The output of this step is represented as ${out}_{3}$, as shown in Equation (13).

(13) $${out}_{3}={PointwiseConv}_{2}(Relu(BN({out}_{2}))$$

After batch normalization, the upgraded feature map is input into the ELA module for local feature enhancement, enhancing the expressive power of disease and pest features. Afterwards, the enhanced feature map is added to the original input x (or $Downsample\left(x\right)$) through residual connections, and the final feature map is output through the relu activation function, as shown in Equation (14), where s represents the step size of group convolution, I is the FLB’s input channel, and O is the FLB output channel. The down-sample consists of a 1 × 1 convolution and batch normalization, as shown in Equation (15).

(14) $$out=\left\{\begin{array}{cc} Relu\left(ELA\left({out}_{3}\right)+x\right), & s=1\;\mathrm{and}\;I=O\\ Relu\left(ELA\left({out}_{3}\right)+Downsample\left(x\right)\right), & s\neq 1\;or\;I\neq O \end{array}\right.$$

(15) $$Downsample\left(x\right)=BN({Conv}_{1\times 1}(x))$$

The FLB module effectively solves the shortcomings of traditional bottleneck modules in dealing with complex background interference by combining pointwise convolution, grouped convolution, the ELA attention mechanism, and residual connections. This design enhances the model’s generalization ability for key features, improving the accuracy and robustness of the model in actual disease and pest identification.

#### 3.2.4. Multi-Hop Local-Feature Fusion Architecture (MLFFA)

Although the basic network architecture of ResNext50 performs well in many visual identification tasks, its performance may not be as good as those specially designed to capture subtle features in disease and pest identification. To capture these subtle features, it may be necessary to adopt more complex feature fusion strategies or adopt deeper or wider network architectures to ensure the effective integration and utilization of features at different scales and levels. In response to the shortcomings of ResNext50 in extracting and fusing fine features of maize diseases and pests, this study proposes a new network architecture, multi-hop local-feature fusion architecture (MLFFA), to replace the original architecture of ResNext50. The design of MLFFA aims to enhance the model’s ability to extract and fuse subtle features of diseases and pests, as shown in Figure 3E.

A new efficient local convolution module, ELC (efficient local convolution), has been introduced in MLFFA for the efficient extraction of subtle features. The ELC model consists of two parts: convolution and ELA. The input is first convolved, and then the output result is enhanced with local features through ELA, as shown in Equation (16), where $x^{\prime }$ is the convolutional output result, GN is group normalization [31], $x_{h}$ and $x_{w}$ are the vertical and horizontal components of the input feature map, σ is the sigmoid activation function, and y is the ELC output result.

(16) $$y=(x^{\prime }\times \sigma (GN({Conv}_{1d}(x_{h})))\times \sigma (GN({Conv}_{1d}(x_{w})))$$

After down-sampling through the max pooling layer, the image is simultaneously feature-extracted through Layer1 and ELC1, and the output results of both are added, element by element, as shown in Equation (17). Next, after batch normalization, ${out}_{1}$ is used as input to Layer2 and ELC2.

(17) $${out}_{1}={out}_{layer1}+{out}_{ELC1}$$

The ${out}_{2}$ output is obtained by adding the outputs of Layer2 and ELC2, as well as the outputs of ${out}_{ELC1}$ after 3 × 3 convolution down-sampling and channel transformation, as shown in Equation (18). Afterwards, ${out}_{2}$ is batch normalized as input for Layer3 and ELC3.

(18) $${out}_{2}={out}_{layer2}+{out}_{ELC2}+{Conv}_{3\times 3}({out}_{ELC1})$$

Similarly, ${out}_{3}$ is obtained by adding the outputs of Layer3 and ELC3, as well as the outputs of ${out}_{ELC2}$ and ${Conv}_{3\times 3}\left({out}_{ELC1}\right)$ after 3 × 3 convolution down-sampling and channel transformation, as shown in Equation (19).

(19) $${out}_{3}={out}_{layer3}+{out}_{ELC3}+{Conv}_{3\times 3}\left({out}_{ELC2}\right)+{Conv}_{3\times 3}({Conv}_{3\times 3}\left({out}_{ELC1}\right))$$

Finally, after batch normalization, ${out}_{3}$ is used as the input to Layer4, and dimensionality is reduced through an average pooling layer. It is then converted into a one-dimensional vector through a flattening layer and finally sent to a fully connected layer for the output of image identification results.

By replacing the original network architecture of ResNext50 with the MLFFA, the input image can obtain more effective subtle feature extraction and fusion, thereby significantly improving the identification accuracy of maize diseases and pests. The MLFFA provides a new, subtle feature extraction solution for disease and pest identification tasks.

## 4. Experimentation and Analysis

This section demonstrates the effectiveness of LFMNet in the identification of 12 types of maize leaf diseases and pests through experiments. The section is divided into eight subsections: (1) Experimental Environment and Preparation; (2) Evaluation Indicators; (3) Performance Analysis of LFMNet; (4) Effectiveness Analysis of Individual Modules; (5) Ablation Experiment; (6) Comparison with Other Advanced Networks; (7) Confusion Matrix of Model Test Results; (8) Model Visualization of LFMNet; (9) Generalization Experiments. We conducted comprehensive performance tests on the model and compared LFMNet with other models from various perspectives.

### 4.1. Experimental Environment and Preparation

To ensure the reliability of the experimental results and avoid the impact of changes in experimental conditions, all experiments in this study were conducted under the same hardware and software conditions. The hardware used includes an NVIDIA GeForce RTX 4090 GPU (NVIDIA, Santa Clara, CA, USA) and an Intel (R) Xeon (R) Platinum 8336C CPU (Intel, Santa Clara, CA, USA) at 2.30 GHz. The server runs on the Windows 10 64-bit operating system. LFMNet was built on the PyTorch 2.1.0 framework. Table 5 provides specific hardware and software details.

The input images are 256 × 256 pixels, and the total number of images is 19,451. The images were divided into a training set of 13,616 images, a validation set of 3889 images, and a test set of 1946 images, with a ratio of approximately 7:2:1. The training set is used for model training, the validation set is used for adjusting model parameters and training parameters, as well as evaluating model performance, and the test set is used for the final assessment of model performance. After continuous adjustments, the final parameter settings during the training process are presented in Table 6.

### 4.2. Evaluation Indicators

In this study, we evaluate the performance of the model using the following metrics: accuracy, precision, recall, F1 score, and testing time.

$$Accuracy=\frac{TP+TN}{TP+TN+FP+FN}\times 100\%$$

$$Precision=\frac{TP}{TP+FP}\times 100\%$$

$$Recall=\frac{TP}{TP+FN}\times 100\%$$

$$F1\;score=\frac{2\times TP}{2\times TP+FP+FN}\times 100\%=2\times \frac{Precision\times Recall}{Precision+Recall}\times 100\%$$

Among them, TP (true positive) represents the number of positive samples correctly predicted by the model, FP (false positive) represents the number of negative samples incorrectly predicted as positive, FN (false negative) represents the number of positive samples incorrectly predicted as negative, and TN (true negative) represents the number of negative samples correctly predicted. Precision measures the proportion of actual positive categories predicted by the model. Recall measures the proportion of all actual positive categories correctly predicted by the model as positive categories. Accuracy measures the proportion of correct predictions among all predictions made by the model. The F1 score is the harmonic mean of precision and recall, which strikes a balance between the two. In image identification, these indicators provide methods for evaluating model performance from different perspectives. Testing time is used to measure the time required for the model, with trained weights, to conduct performance testing on the test set.

### 4.3. Performance Analysis of LFMNet

**Figure 4:**
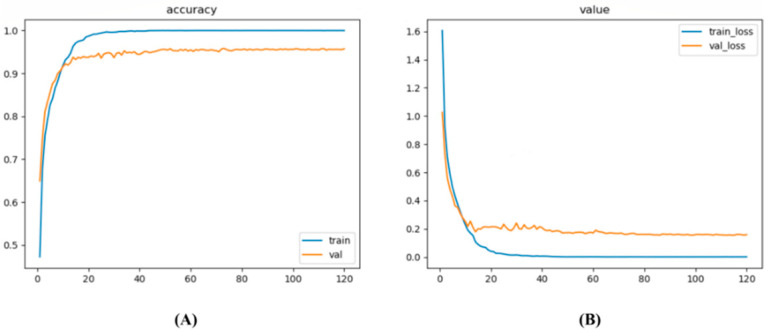
Changes in accuracy and loss value during the training process of LFMNet. (**A**) Accuracy variation curve. (**B**) Loss value variation curve.

In order to evaluate the performance of LFMNet in identifying 12 types of maize leaves, we conducted training and validation on the MLDP dataset. We initially trained the model, adjusting the training parameters based on the metrics of the validation set during the training process. The accuracy and loss values for both the training and validation sets were recorded and plotted in Figure 4, which reflects the model’s generalization ability on the dataset to some extent. As depicted in the graph, after training for 120 epochs, the accuracy of the training process reached 99.99%, and the loss decreased to around 0.0009. The accuracy of the validation process reached 95.78%, and the loss decreased to around 0.1548.

**Figure 5:**
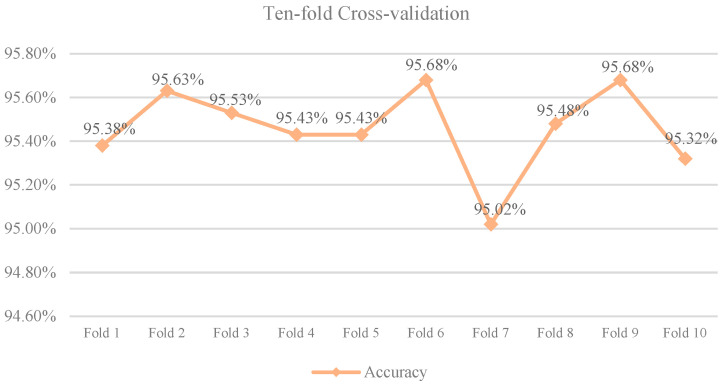
Ten-fold Cross-Validation of LFMNet on the MLDP Dataset.

To minimize experimental variability, we conducted a ten-fold cross-validation [34] on LFMNet. In this procedure, the dataset was divided into 10 subsets, with each subset alternately serving as the test set, while the remaining 9 subsets were used as the training set. Subsequently, LFMNet underwent training and testing in each iteration, and the results of the ten-fold cross-validation were aggregated. The summarized accuracy for the ten-fold cross-validation is presented in Figure 5, with an average accuracy of 95.46%.

### 4.4. Effectiveness Analysis of Individual Modules

#### 4.4.1. Effectiveness of LMSB

In this study, we propose a new initial down-sampling block, the LMSB, aimed at solving the problem of traditional 7 × 7 convolution losing important feature information during initial down-sampling. To verify the effectiveness of the LMSB, we compared it with the ResNet-C [35] module. The experimental results are shown in Table 7.

The ResNet-C module adopts the strategy of replacing 7 × 7 convolutions with three consecutive 3 × 3 convolutions. The first 3 × 3 convolution is responsible for down-sampling the image, while the next two 3 × 3 convolutions further extract the features of the feature map. However, although this design can provide more detailed feature extraction in some cases, it comes at the cost of increasing the overall number of parameters, and its performance in maize disease and pest identification tasks is not ideal. This indicates that the ResNet-C module may be more suitable for application scenarios that do not require high requirements for fine feature extraction.

In contrast, the LMSB utilizes its multi-scale and feature localization characteristics to effectively preserve important feature regions in the image while down-sampling. This is crucial for capturing subtle features in subsequent network structures. The design of the LMSB helps to improve the extraction effect of disease and pest features but also maintains a lower number of parameters, which is a significant advantage in model design. The experimental results, as shown in Table 7, demonstrate that the LMSB can provide better performance compared to ResNet-C while maintaining parameter efficiency, especially in maize disease and pest identification tasks that require high-precision feature extraction.

#### 4.4.2. Effectiveness of FLB

In this study, to address the shortcomings of the bottleneck module in handling complex background interference and fine feature extraction, we proposed an FLB. To verify the effectiveness of the FLB, we conducted comparative experiments with three existing bottleneck block improvement schemes: CBAMB, composed of CBAM; CAB, composed of CA; SEB, composed of SE; and the original bottleneck. The experimental results are shown in Table 8.

The CBAMB achieves dual optimization of the bottleneck structure by integrating the CBAM attention mechanism, enabling the network to focus on information-rich channels and regions in the image, and improving the model’s ability to recognize image features. However, the spatial attention mechanism of CBAMB is two-dimensional and not as flexible and detailed as ELA in FLB, which may limit the performance of CBAMB in tasks that require subtle spatial feature identification. The CAB integrates the CA (coord attention) attention mechanism to bring dynamic focusing ability in the spatial dimension to the module, improving the accuracy and efficiency of image identification. However, although the component CA attention mechanism of the CAB enhances coordinate information, it also reduces channel dimension, which has a negative impact on the generated attention and limits the performance of the CABottleneck. The SEBottleneck introduces the SE (squeeze and excitation) attention mechanism to achieve adaptive weighting of channel features in the bottleneck structure, improving the ability of feature representation and enhancing the accuracy and generalization ability of the network. The SE attention mechanism of SEB mainly focuses on the channel dimension, capturing statistical information between channels through global average pooling, but lacks attention in the spatial dimension.

Compared with the above scheme, the ELA attention mechanism in the FLB independently applies attention in two dimensions of space (height and width), which can more finely adjust the spatial features of the feature map. According to the experimental results in Table 8, the FLB is superior to the CBAMB, CAB, and SEB in all indicators. Therefore, we chose the FLB as the basic component of LFMNet to achieve significant improvements in its complex background interference processing ability and fine-feature extraction ability. The design of the FLB considers the fine extraction of spatial features, which is crucial for improving the performance of the model in practical disease and pest identification tasks. By introducing the FLB, LFMNet can more effectively identify diseases and pests on maize leaves, maintaining high accuracy even in complex and variable backgrounds.

#### 4.4.3. Effectiveness of the MLFFA

This study proposes the MLFFA with the aim of addressing the limitations of ResNext50 in extracting the subtle features of maize leaf diseases and pests. The design core of MLFFA lies in its multi-hop local-feature fusion architecture, which enables the network to more effectively capture and integrate the subtle features of diseases and pests. To verify the effectiveness of the MLFFA, we compared it with the original ResNext50 architecture and the ResNet50 architecture.

The experimental results showed that the MLFFA has significant advantages in capturing subtle features of maize leaf diseases and pests and exhibits more accurate and robust performance in disease and pest identification tasks compared to ResNext50 and ResNet50. The specific experimental results are shown in Table 9. In terms of various evaluation indicators, the MLFFA is superior to the ResNext50 architecture and the ResNet50 architecture. Based on these results, we adopt the MLFFA as the network architecture in LFMNet to achieve the effective extraction and fusion of fine features of diseases and pests.

### 4.5. Ablation Experiment

This paper proposes the LMSB, the FLB, and the MLFFA. To evaluate the effectiveness of these modules, we conducted ablation experiments. In this context, the LMSB is the localized multiscale inverted residual convolutional block proposed in this paper, the FLB is the feature localization bottleneck proposed in this paper, and the MLFFA is the multi-hop local-feature fusion architecture. We used the control variable method to gradually replace the LMSB, FLB, and MLFFA in the ResNext50 model, forming seven different schemes. The models corresponding to each scheme are trained using unified training parameters on MLDPs to ensure the fairness of the experiment. The training parameters are shown in Table 6 of Section 4.1. After completing the training, all models were tested on the same test set, and the final test results are summarized in Table 10. The experimental results show that, while keeping other conditions the same, the identification accuracy of the model gradually improves with the integration of new modules. This discovery validates the effectiveness of the LMSB, FLB, and MLFFA in improving model performance.

Through these experiments, we not only demonstrated the independent contributions of each module but also showed that, when they work together, they can significantly improve the accuracy of identifying maize leaf diseases and pests.

### 4.6. Comparison with Other Advanced Networks

**Figure 6:**
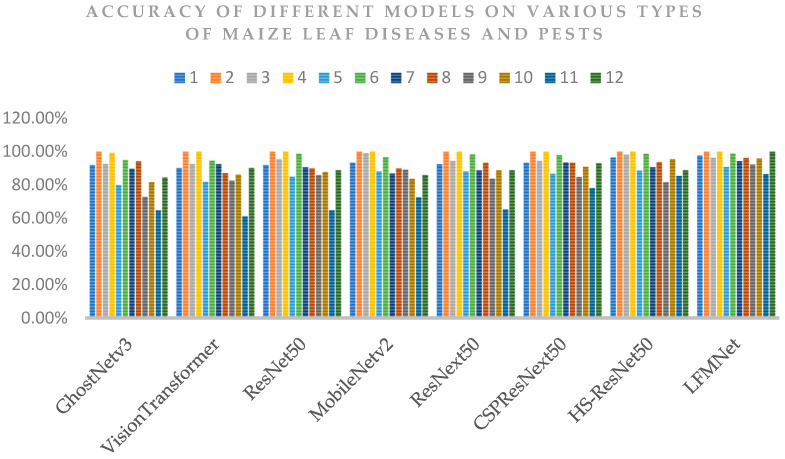
Accuracy of different models on various types of maize leaves.

We compared LFMNet with other advanced networks in this study. The networks used for comparison included CSPResNext50 [39], ResNet50 [40], ResNext50, MobileNetv2 [41], HS-ResNet50 [42], GhostNetv3 [43], and Vision Transformer [44]. The training parameters of all models are the same, as shown in Table 6. All models were trained for 120 epochs, and testing was conducted on the same test set. The experimental results are presented in Table 11. Afterwards, we conducted statistical analysis on the accuracy of each model on each type of maize leaf image, as illustrated in Figure 6. Here, numerical values are used to represent each type of maize leaf in place of their complete names: 1 blight; 2 common rust; 3 gray leaf spot; 4 healthy; 5 leaf beetle; 6 mold; 7 northern leaf blight; 8 phosphorus deficiency; 9 potassium deficiency; 10 red spider mite; 11 mythimna separata (walker); 12 phyllotreta striolata.

From Table 11, it is evident that LFMNet achieves the highest accuracy, precision, recall, and F1 score among the eight models. Figure 6 shows that LFMNet achieves the highest accuracy in identifying 12 different types of leaves, except for the third type, where the identification accuracy is slightly lower than the highest accuracy. This result highlights the robustness and accuracy of the LFMNet model in identifying diverse diseases and pests in maize leaves.

### 4.7. Confusion Matrix of Model Test Results

**Figure 7:**
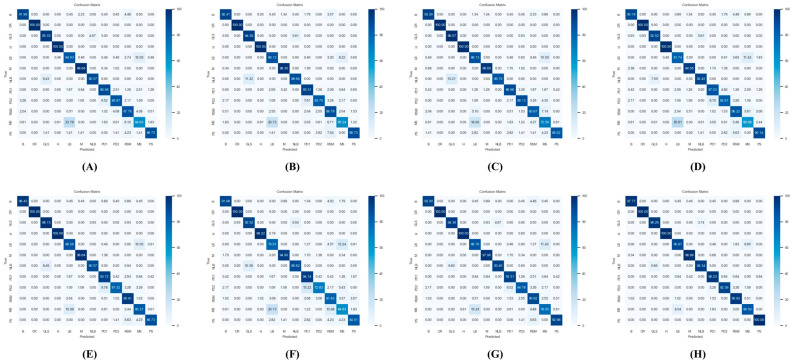
Confusion matrix of model test results: (**A**) ResNet50, (**B**) ResNext50, (**C**) MobileNet-v2, (**D**) Vision Transformer, (**E**) HS-ResNet50, (**F**) GhostNet-v3, (**G**) CSPResNext50, and (**H**) FLMNet.

The confusion matrix between LFMNet and other models is shown in Figure 7. The subfigures represent the following: (A) ResNet50, (B) ResNext50, (C) MobileNet-v2, (D) Vision Transformer, (E) HS-ResNet50, (F) GhostNet-v3, (G) CSPResNext50, and (H) LFMNet. The following is an explanation of the abbreviations of leaf names in the confusion matrix: B represents *blight*, CR represents *common rust*, GLS represents *gray leaf spot*, H represents *healthy*, LB represents *leaf beetle*, M represents *mold*, NLB represents *northern leaf blight*, PD1 represents *phosphorus deficiency*, PD2 represents *potassium deficiency*, RSM represents *red spider mite*, MS represents *mythimna separata* (*walker*) and PS represents *phyllotreta striolata*.

Through the confusion matrix, we can clearly see the advantages of LFMNet in identifying various maize leaf diseases and pests.

### 4.8. Model Visualization of LFMNet

**Figure 8:**
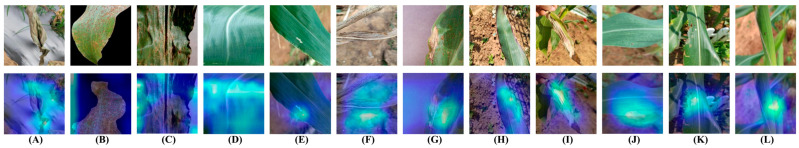
Model visualization of LFMNet. From (**A**) to (**L**), 12 types of maize leaves are represented in sequence: (**A**) blight, (**B**) common rust, (**C**) gray leaf spot, (**D**) healthy, (**E**) leaf beetle, (**F**) mold, (**G**) northern leaf blight, (**H**) phosphorus deficiency, (**I**) potassium deficiency, (**J**) red spider mite, (**K**) mythimna separata (walker), (**L**) phyllotreta striolata.

In order to visually observe the impact of different regions of different types of maize leaves on LFMNet for disease and pest identification, we visualized some images using the model weights obtained after training. We used Grad-CAM to visualize the weights of the LFMNet model. Figure 8 shows the visualization result generated by overlaying the maize leaf image with its thermal map.

The visualization results shown in Figure 8 clearly demonstrate the excellent performance of LFMNet in extracting subtle features of maize leaf diseases and pests. By overlaying the heatmap, we can intuitively see how the model effectively focuses on key feature areas in the image. These areas are often the decisive factors for identifying diseases and pests, and LFMNet can accurately identify and give them more attention, significantly improving the accuracy of identification.

### 4.9. Generalization Experiments

Generalization experiments are crucial for evaluating the practical application potential of models. They test the applicability and robustness of models by training and testing them on diverse datasets. These experiments particularly focus on the performance of the model under various input conditions, as well as on its ability to accurately identify target objects (such as plant leaves) in complex backgrounds. Through this comprehensive testing, we can verify whether the model has the ability to maintain consistent performance in a wide range of scenarios, not just based on specific training data. The purpose of generalization experiments is to ensure that the model can overcome the limitations of the training environment and demonstrate stable predictive ability in real-world applications. A comprehensive generalization evaluation not only helps us gain a deeper insight into the generalization potential of the model but also provides us with an important basis for judging the reliability of the model. In addition, it also reveals the problems and limitations that may be encountered during the actual deployment process, thereby pointing the way for further optimization and improvement of the model.

In order to comprehensively evaluate the generalization ability of the LFMNet model, we selected four standard datasets for experiments, including Plant Village-Apple, Almond, Fish, and Plant Leaves, as shown in Table 12. These datasets cover different fields, which can comprehensively test the applicability of the model in various scenarios. We also implemented data augmentation strategies for the Plant Village-Apple and Almond datasets to further test the robustness of the model when facing diverse data. The training parameters are shown in Table 6 of Section 4.1. All datasets are divided into training and testing sets, with an approximate ratio of 9:1. The experimental results show that LFMNet exhibits excellent performance on all four datasets. Specifically, as shown in Table 13, the accuracy on the Plant Village-Apple, Almond, Fish, and Plant Leaves datasets reached 99.37%, 99.35%, 99.67%, and 99.44%, respectively. These results not only demonstrate the accuracy of LFMNet in identifying maize leaf diseases and pests but also highlight its cross-domain generalization ability.

The generalization experiment has demonstrated that LFMNet not only performs well in identifying maize leaf diseases and pests but also maintains high accuracy in identifying different types of image data. This indicates that LFMNet has good generalization potential and can play an important role in practical applications.

## 5. Discussion

We propose an identification method for maize leaf diseases and pests based on LFMNet and verify the effectiveness of this method through a series of experiments. Table 11 and Figure 6 show that LFMNet has improved accuracy and precision in 12 maize-leaf classification tasks compared to other existing models. 

GhostNet v3 is centered around its lightweight design, providing efficient computational performance, even while maintaining a small model size. However, on the MLDP dataset, GhostNet v3 did not demonstrate its advantages, with accuracy, precision, recall, and F1 scores of 87.62%, 87.49%, 87.16%, and 87.25%, respectively, and a testing time of 7.78 s. Vision Transformer divides an image into multiple small blocks and linearly embeds them into a sequence, allowing the transformer to process the image similarly to a text sequence. However, on the MLDP dataset, Vision Transformer did not perform well, with accuracy, precision, recall, and F1 scores of 87.72%, 87.16%, 88.20%, and 87.59%, respectively, and a testing time of 7.27 s. ResNet50, combined with residual blocks, achieved an accuracy of 89.88%, an accuracy of 89.63%, a recall rate of 89.86%, an F1 score of 89.68%, and a testing time of 3.47 s on the 12 types of maize leaves. MobileNetv2 is a lightweight deep neural network architecture with a testing time of 3.31 s while maintaining high performance, achieving an accuracy of 90.39%, a recall of 90.43%, and an F1 score of 90.56%. ResNext50 improves the model’s representation ability by introducing the concept of group convolution on the basis of ResNet50. Compared to ResNet50, ResNext50 achieved higher performance with a comparable number of parameters, specifically with an accuracy of 90.54% and a testing time of 3.56 s. In addition, ResNext50 also performed well in accuracy, recall, and the F1 score, with scores of 90.81%, 90.14%, and 90.36%, respectively. CSPResNext50 is an efficient convolutional neural network architecture that combines the computational efficiency of Cross-Stage Partial Network (CSPNet) with the feature representation capability of ResNext50, achieving an accuracy of 92.19%, an accuracy of 92.45%, a recall rate of 92.14%, an F1 score of 92.28%, and a testing time of 4.86 s. HS ResNet50 is an improved ResNet model that enhances the feature extraction ability of the model by introducing a hybrid shift module, achieving an accuracy of 93.73%, an accuracy of 94.04%, a recall rate of 93.09%, and an F1 score of 93.49%. However, the testing time of HS ResNet50 is relatively long, reaching 21.59 s, which may become a limitation in practical applications. LFMNet achieved excellent performance with an accuracy of 95.68%, an accuracy of 95.91%, a recall rate of 95.78%, an F1 score of 95.83%, and a testing time of 4.71 s. Compared to ResNext50, LFMNet improved accuracy, precision, recall, and F1 score by 5.14%, 5.1%, 5.64%, and 5.47%, respectively, while also increasing testing time by 1.15 s. 

Figure 7 shows the confusion matrix of eight models. The confusion matrix is an important tool for evaluating the performance of classification models. It presents the relationship between model predictions and actual labels in an intuitive table form, identifies misclassifications of the model in specific categories, guides model optimization, and provides more accurate performance evaluations in cases of imbalanced categories. The confusion matrix shows that LFMNet still has room for improvement in the identification of gray leaf spot and *Mythimna separata* (walker), two types of maize leaf diseases. The identification accuracy for gray leaf spot is 2.81%, lower than that of MobileNetv2. Although the highest identification accuracy among the eight models was achieved on the *Mythimna separata* (walker), it only reached 86.59%.

The following is a summary of the factors that improve the performance of the LFMNet model:

(1) The LMSB module proposed in LFMNet accurately preserves important disease and pest feature regions during initial down-sampling, which helps to extract fine features from subsequent network structures. According to Table 7, LMSB has a positive impact on all four key identification indicators. In Table 10, schemes D, E, and G are compared to schemes B, C, and F. The accuracy increased by 0.41%, 1.90%, and 0.95%, respectively, demonstrating the contribution of LMSB to performance improvement.
(2) To cope with complex background interference, LFMNet adopts the FLB components. Compared to bottleneck, CBAMB, CAB, and SEB, the FLB showed an accuracy improvement of 2.45%, 1.34%, 1.80%, and 1.96% in Table 8. In Table 10, schemes D, F, and G are compared to schemes A, C, and E. The accuracy was improved by 3.08%, 2.62%, and 2.69%, respectively, further confirming the role of the FLB in improving the performance of LFMNet.
(3) In response to the challenge of extracting subtle features, the MLFFA module proposed in this study replaces the ResNext50 architecture. Table 9 shows that compared to ResNet50 and ResNext50, the MLFFA achieved an accuracy improvement of 2.41% and 1.73%, respectively. In Table 10, schemes E, F, and G are compared to schemes A, B, and D. The accuracy has increased by 2.67%, 0.72%, and 1.28%, respectively, fully demonstrating the effectiveness of the MLFFA.

The LFMNet model has shown excellent performance in identifying maize leaf diseases and pests, not only achieving high-precision recognition on specific datasets but also demonstrating good generalization ability on other datasets. This achievement can improve the automation level of identifying maize leaf diseases and pests, effectively supplementing the shortcomings of traditional manual identification methods. 

However, in practical applications, in order to meet the needs of real-time processing, we recognize the need to further optimize the model parameters. This includes improving the computational speed of the model without sacrificing recognition accuracy to ensure that LFMNet can respond quickly in actual agricultural production environments. Meanwhile, the same diseased leaves will exhibit different degrees of disease, such as early, middle, and late stages. Leaves with different degrees of disease may appear completely different in appearance. It is necessary to further classify diseased leaves based on their degree of illness.

In the future, we plan to further classify the same diseased leaves according to their severity in order to improve the reliability of our research. We also plan to make lightweight improvements to LFMNet to enhance its applicability in real-time application scenarios. In addition, current research mainly focuses on the identification of pests and diseases during the image acquisition stage. In the future, we will expand our research scope and apply this technology to every step of the maize planting cycle, thereby providing more comprehensive and in-depth technical support for agricultural production.

## 6. Conclusions

The LFMNet model based on the ResNext50 architecture proposed in this study significantly improves the accuracy and robustness of maize leaf pest identification by introducing innovative modules such as the LMSB, FLB, and MLFFA. The experimental results on the self-built dataset showed that the average accuracy of LFMNet reached 95.68%, with an F1 score of 95.83%, proving its excellent performance. In addition, through comparison with other models and testing its generalization ability, LFMNet has demonstrated its superiority in image recognition tasks. This achievement is expected to improve the automation level of disease and pest identification, effectively supplementing the shortcomings of traditional manual identification methods.

However, in order to meet the demand for real-time processing, we recognize the need to further optimize model parameters to improve recognition speed and maintain accuracy, ensuring that LFMNet can respond quickly in actual agricultural production. Meanwhile, considering the appearance differences of the same disease at different stages, we plan to conduct more detailed leaf classification based on disease severity in future research to enhance the reliability of the study. In addition, we will expand our research scope and apply this technology to various stages of maize cultivation, providing more comprehensive technical support for agricultural production.

## Figures and Tables

**Table 1:** Leaf characteristics of maize affected by diseases and pests.

Disease and Pest Category	Characteristics of Leaves
Blight	The leaves are grayish green in color, with a shape resembling hot water or frost.
Common rust	Orange-brown abscesses appear on the leaves, which, in severe cases, cover the entire leaf surface.
Gray leaf spot	The initial appearance of the lesion is surrounded by a yellow halo, which later forms rectangular gray spots parallel to the leaf veins.
Mold	Some areas of the leaves exhibit black mold spots.
Northern leaf blight	Thin grayish-green to brownish-brown lesions appear on the leaves.
Phosphorus deficiency	The leaf tips and edges appear purplish-red.
Potassium deficiency	Yellow stripes appear from the leaf tips towards the base of the leaves, with the tips and edges turning yellow.
Leaf beetle	Leaves with residual reticular veins or epidermis appear as small, irregular white spots from a distance.
Red spider mite	The surface of the leaves is covered with flocculent or mesh-like substances.
*Mythimna separata* (walker)	The leaves exhibit holes, or irregular notches.
*Phyllotreta striolata*	Adults with yellow stripe markings can be seen on the leaves.

**Table 2:** Images of maize leaf diseases and pests.

Diseases and Pests	Images		
Gray leaf spot	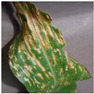	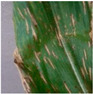	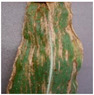
Common rust	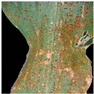	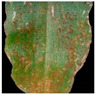	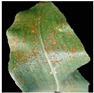
Northern leaf blight	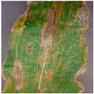	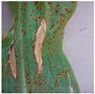	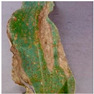
Blight	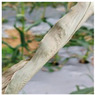	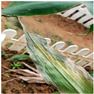	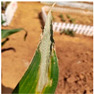
Mold	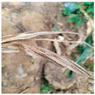	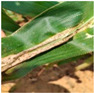	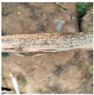
Phosphorus deficiency	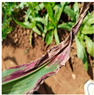	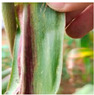	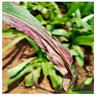
Potassium deficiency	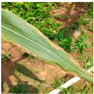	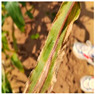	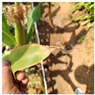
Leaf beetle	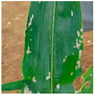	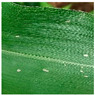	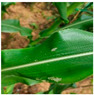
Red spider mite	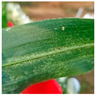	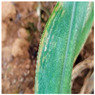	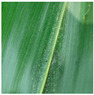
*Mythimna separata* (walker)	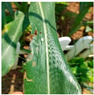	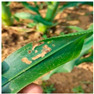	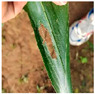
*Phyllotreta striolata*	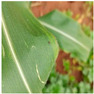	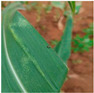	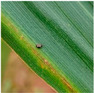

**Table 3:** Distribution of various maize leaf images in the initial dataset.

Disease or Pest Category	Number	Proportion
Blight	2148	11.88%
Common rust	1192	6.59%
Gray leaf spot	513	2.84%
Healthy	1162	6.43%
Leaf beetle	2215	12.25%
Mold	3141	17.37%
Northern leaf blight	985	5.45%
Phosphorus deficiency	2391	13.22%
Potassium deficiency	478	2.64%
Red spider mite	2011	11.12%
*Mythimna separata* (walker)	1463	8.09%
*Phyllotreta striolata*	383	2.12%

**Table 4:** Number of various images in the MLDP dataset after data augmentation.

Disease or Pest Category	Number	Proportion
Blight	2148	11.04%
Common rust	1192	6.13%
Gray leaf spot	1025	5.27%
Healthy	1162	5.97%
Leaf beetle	2215	11.39%
Mold	3140	16.14%
Northern leaf blight	985	5.06%
Phosphorus deficiency	2391	12.29%
Potassium deficiency	954	4.91%
Red spider mite	2011	10.34%
*Mythimna separata* (walker)	1463	7.52%
*Phyllotreta striolata*	765	3.94%

**Table 5:** Software and hardware environments.

Hardware environment	CPU	Intel(R) Xeon(R) Platinum 8336C
	GPU	NVIDIA GeForce RTX 4090
Software environment	CUDA	11.8
	OS	Windows 10 64-bit
	Pycharm	2023.2.1
	Python	3.11.5
	Pytorch	2.1.0

**Table 6:** Experimental parameter settings.

Parameter	Numerical Value
Initial learning rate	2 × 10^−5^
Batch size	16
Number of iterations	120
Optimizer	SGD
Weight decay	5 × 10^−4^
Momentum	0.999
Lr scheduler	CosineAnnealingLR
T_max	120

**Table 7:** Comparison of the capabilities of three initial down-sampling blocks under the same conditions.

Methods	Precision	Recall	F1	Accuracy	Parameters
Conv	90.81	90.14	90.36	90.54	12.923M
ResNet-C	87.25	86.82	87.02	87.31	12.989M
LMSB	91.26	91.60	91.34	91.32	12.928M

**Table 8:** Comparison of five bottleneck abilities under the same condition.

Methods	Precision	Recall	F1	Accuracy
Bottleneck	90.81	90.14	90.36	90.54
CBAMB [36]	92.72	92.48	92.56	92.65
CAB [37]	92.28	91.98	92.09	92.19
SEB [38]	92.17	91.98	92.03	92.03
FLB	94.05	93.92	93.93	93.99

**Table 9:** Comparison of the three architectures’ abilities under the same condition.

Methods	Precision	Recall	F1	Accuracy
ResNet50	89.88	89.63	89.86	89.68
ResNext50	90.54	90.81	90.14	90.36
MLFFA	92.35	92.20	92.26	92.09

**Table 10:** Ablation experiment.

Schemes	Methods	Precision	Recall	F1 Score	Accuracy
A	LMSB	91.26	91.60	91.34	91.32
B	FLB	94.05	93.92	93.93	93.99
C	MLFFA	92.35	92.20	92.26	92.09
D	LMSB + FLB	94.46	94.27	94.35	94.40
E	LMSB + MLFFA	94.07	93.94	93.93	93.99
F	FLB + MLFFA	94.84	94.71	94.75	94.71
G	LMSB + FLB + MLFFA	95.91	95.78	95.83	95.68

**Table 11:** Comparison with other advanced networks.

Network	Accuracy (%)	Precision (%)	Recall (%)	F1 Score (%)	Testing Time (s)
GhostNet-v3	87.62	87.49	87.16	87.25	7.78
Vision Transformer	87.72	87.16	88.20	87.59	7.27
ResNet50	89.88	89.63	89.86	89.68	3.47
MobileNet-v2	90.39	90.97	90.43	90.56	3.31
ResNext50	90.54	90.81	90.14	90.36	3.56
CSPResNext50	92.19	92.45	92.14	92.28	4.86
HS-ResNet50	93.73	94.04	93.09	93.49	21.59
LFMNet	95.68	95.91	95.78	95.83	4.71

**Table 12:** Public datasets in the generalization experiments.

Dataset	Total	Category	Available
Plant Village-Apple	8227	4	https://www.kaggle.com/datasets/hiyash99/plantvillage (accessed on 2 June 2024)
Almond [45]	3694	4	https://www.kaggle.com/datasets/mahyeks/almond-varieties (accessed on 2 June 2024)
Fish [46]	8980	9	https://www.kaggle.com/datasets/crowww/a-large-scale-fish-dataset (accessed on 2 June 2024)
Plants Leaves	17,727	14	https://www.kaggle.com/datasets/manuelcecerepalazzo/leaves-images (accessed on 2 June 2024)

**Table 13:** Results of the generalizability experiments.

Dataset	Accuracy (%)	Precision (%)	Recall (%)	F1 Score (%)
Plant Village-Apple	99.37	99.37	99.37	99.37
Almond	99.35	99.19	99.42	99.30
Fish	99.67	99.66	99.64	99.65
Plant leaves	99.44	99.63	99.37	99.50

## Data Availability

The original contributions presented in the study are included in the article. Further inquiries can be directed to the corresponding authors.
